# Clinicopathological features and reclassification of penile squamous cell carcinoma according to WHO classification 2022 for penile carcinoma with p16 immunohistochemical expression and its prognostic impact

**DOI:** 10.1186/s13000-025-01676-5

**Published:** 2025-07-03

**Authors:** Vaanya Kaushik, Kanthilatha Pai, Anuradha Rao, Swati Sharma

**Affiliations:** Dept of Pathology, Kasturba Medical College, Manipal Academy of Higher Education, Manipal, 576104 India

**Keywords:** Penile squamous cell carcinoma, HPV, p16 immunohistochemistry, WHO classification

## Abstract

**Introduction:**

Squamous cell carcinoma (SCC) is the most common type of penile cancer, and infection with human papillomavirus (HPV) is one of the most highly associated risk factors. The WHO classification for penile SCC (2022) strongly recommends and advocates penile SCC to be reported as HPV-associated or HPV-independent type in pathology reports. Further, p16 immunohistochemistry (IHC) is recommended to classify SCC into the above major types, although it is not completely reliable for HPV infection. Although there are no established differences in the prognosis or treatment between HPV-associated and HPV-independent penile tumours, there is recent evidence to suggest that HPV-associated SCC may respond better to radiation therapy, immunotherapy, etc.

**Aim and objectives:**

This study aims to = analyse the clinicopathological features of penile squamous cell carcinoma and reclassify penile SCC into HPV-associated and HPV-independent types to align with the WHO classification of penile carcinoma (2022) and study the expression of p16 by immunohistochemistry. Additionally, we studied the prognostic significance of HPV-associated and independent SCC based on histology and p16 immunostaining.

**Materials and methods:**

This is a five-year retrospective single-institution study that included all diagnosed cases of penile SCC. Clinicopathological features and p16 expressions were studied and analysed.

**Results:**

A total of 72 cases of penile SCC were included during the study period. The mean age of occurrence of penile SCC was 58 years. The most common site of the tumor was the glans penis (50.74%). We encountered only 6 cases (8.3%) of HPV-associated type of penile SCC, while the majority belonged to the HPV-independent type (91.7%) based on histology. p16 immunohistochemistry showed positivity in 15 cases (21%) and negativity in 57 cases (79%). Most of the tumors showed favorable features– histological grade I, pathological T1 stage with a low incidence of nodal metastasis. There was a strong association between histological subtyping into HPV-associated and independent SCC with p16 IHC expression (*p* = 0.015). Classification of penile SCC by histology and p16 expression into HPV associated and independent type showed no prognostic significance with pathological stage but was significant with histological grade and lymph node metastasis.

## Introduction

Penile cancer is a relatively rare form of cancer worldwide. However, its incidence is significantly higher in developing countries, including parts of India, where sociocultural factors, hygiene practices, literacy, and limited access to early healthcare may result in delayed diagnosis and poorer outcomes [1, 2, 3].

The most common type of cancer affecting the penis is squamous cell carcinoma (SCC). Penile SCC is a clinically aggressive cancer with a tendency for loco-regional spread that significantly affects the physical and psychological quality of life of patients. The etiopathogenesis of penile SCC is multifactorial. High-risk human papillomavirus (HPV) infection with HPV 16 and 18 has been implicated in a subset of cases [4–6]. In a largest epidemiological study on HPV Subtypes in penile SCC, HPV 6 was found to be the second most prevalent subtype by Olesen et al. [7] WHO classification 2022 classifies penile cancers into HPV-associated and HPV-independent carcinomas, recognizing the distinct clinical, morphological, and molecular characteristics of these tumors 2.

p16^INK4a^(p16), a cyclin-dependent kinase inhibitor, is commonly used as a surrogate immunohistochemical marker for detecting high-risk HPV infection and is considered a reliable indicator of HPV-driven oncogenesis in several anogenital and head and neck squamous cell carcinomas [8–12].

In the Indian context, there is a lack of comprehensive studies evaluating the prevalence of penile carcinomas, and their classification into HPV and non-HPV types based on morphology and expression of p16-immunohistochemistry. Given the socio-demographic differences and varying HPV prevalence in different parts of the country, it is hence crucial to understand the clinicopathological features of penile SCC and study the role of p16 as a diagnostic and prognostic marker in penile SCC.

The study aims to understand the frequency of HPV-independent and HPV-associated SCC by histology and their p16 immunohistochemical expression and explores the prognostic correlation between these subtypes with stage and other clinicopathological parameters.

## Materials and methods

This study was undertaken to retrospectively evaluate 72 cases of penile SCC diagnosed over a five-year period in the Department of Pathology at a tertiary care center in India, to analyze the clinicopathological features and expression of p16 by immunohistochemistry, after obtaining Institutional Ethics Committee clearance.

The clinical features were obtained from hospital records. Data regarding patient age, circumcision status, history of smoking, sexually transmitted infections, size and location of the lesion, presence of phimosis, inguinal lymphadenopathy, and nature of treatment were noted. Pathological features, including histological type, grade, and pathological staging, were obtained from histopathology reports and by reviewing H&E slides.

The tumors were reclassified into HPV-associated and HPV-independent types according to the WHO Classification (2022) based on morphology and by p16 immunohistochemistry. The 2022 WHO classification of penile squamous cell carcinoma (SCC) divides tumors into HPV-associated and HPV-independent types based on morphology and supported by p16 immunohistochemistry. HPV-associated subtypes include basaloid, warty, clear cell, lymphoepithelioma like carcinoma and mixed that includes warty basaloid and other admixed type. HPV-independent carcinomas include usual-type including pseudohyperplastic and pseudoglandular, papillary, verrucous including carcinoma cuniculatum, sarcomatoid, and mixed types. SCC were graded using a three-tier grading system into Grade I, II, and III as per the AJCC grading criteria. Tumor staging was done according to the AJCC 8th Edition (2018) criteria for penile cancer.

### p16 immunohistochemistry

IHC was performed on all cases using p63 (clone MX007, mouse monoclonal antibody, Master Diagnostica Company). Representative tumor areas were identified and marked on the slides and corresponding formalin-fixed, paraffin-embedded blocks. Manual IHC staining was performed on tumor samples along with appropriate positive control (HPV-positive oropharyngeal squamous cell carcinoma), beginning with heat-induced epitope retrieval. Primary antibody incubation was carried out for one hour, followed by horseradish peroxidase-labelled MACH1 antibody, using Betazoid diaminobenzidine as the chromogen. Slides were counterstained with hematoxylin, and results were interpreted accordingly.

IHC slides were considered positive if tumor cells showed strong, continuous nuclear and cytoplasmic staining of p16 (block positivity) as described in the study by Cubilla and Martin et al. [13, 14] p16 expression was then analyzed in relation to the histological subtype and other pathological parameters.

### Statistical analysis

IBM-SPSS Statistics for Windows (Version 23.0) was used (Armonk, NY: IBM Corp). For categorical variables, percentage analysis was utilized, and for continuous variables, mean and standard deviation were used to explain the data descriptive statistics and frequency analysis. The Chi-Square Test was employed for determination of the significance of categorical data. The probability value (*p* value) of 0.05 was deemed significant in all the statistical tools.

## Results

A total of 72 patients with penile squamous cell carcinoma (SCC) were worked-up. The majority of cases were over 50 years of age (73.6%), while 26.4% were under 50 years. Most patients were married (82%). The most commonly affected site was the glans with or without involvement of shaft ( 63.9%) and the shaft alone in 16.7%. Less commonly involved sites included the coronal sulcus (5.6%), foreskin (5.6%), and root of the penis (2.8%).

Among known risk factors, 80% of patients with a history of smoking (*n* = 15) reported tobacco use. Circumcision was performed in 24% of patients (12/25). Phimosis was present in 37.5% of cases, and 9.7% had a history of balanoposthitis. HIV positivity was observed in 4.2% of the cohort.

Regarding tumor characteristics, 55.4% of tumors were larger than 3 cm, while 44.6% were smaller. Inguinal lymph node metastasis was detected in13.6% (8/59), and distant metastasis was observed in 3.4%. The recurrence rate was 3.4%. The clinical characteristics are summarized in Table 1.

**Table 1:** Shows clinical features of penile SCC

	Number	Percentage
*AGE* < 50 years > 50 years	19 53	26.4% 73.6%
Married	59	82%
*SITE* Coronal sulcus Foreskin Glans Glans and shaft Shaft Root of penis	4 4 34 12 12 2	5.6% 5.6% 47.2% 16.7% 16.7% 2.8%
H/o smoking (*n* = 15)	12	80%
Circumscision (*n* = 25)	6	24%
HIV positive status	3	4.2%
Phimosis	27	37.5%
H/O Balanoposthitis	7	9.7%
Size of the tumor (*n* = 65) < 3 cm > 3 cm	29 36	44.6% 55.4%
Inguinal node metastasis ( *n* = 59)	8	13.6%
Distant metastasis (*n* = 59)	2	3.4%
Recurrence	2	3.4%

Pathological evaluation revealed that the majority of specimens were obtained through partial penectomy (51.4%), followed by total penectomy (25%), small biopsies (18%), and wide local excisions (5.5%).

Based on morphology, the majority belonged to HPV-independent SCC (91.7%), with the usual subtype (including pseudoglandular variant) being the most common, accounting for 76.4% of cases. Verrucous carcinoma, papillary, and sarcomatoid subtypes were seen in 11.1%, 2.8%, and 1.4% of patients, respectively. HPV-associated subtypes based on morphology accounted for a smaller proportion that included basaloid (2.8%), warty (2.8%), mixed warty-basaloid (1.4%), and lymphoepithelioma-like subtypes (1.4%).

**Fig. 1:**
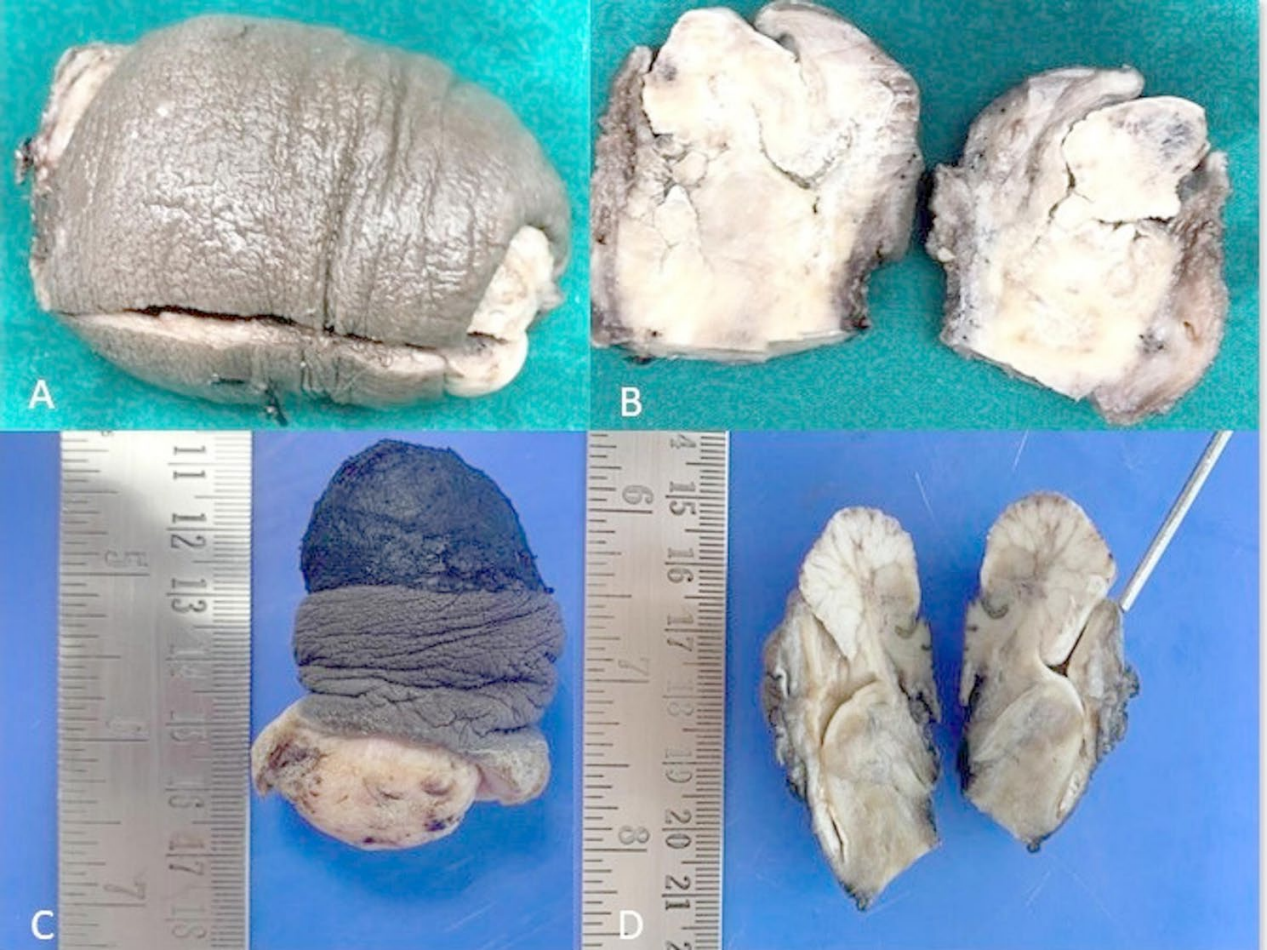
Shows gross images of HPV independent Penile SCC: **A&B**: External and cut section of Usual type of Penile SCC (Carcinoma cuniculatum subtype) **C&D** shows external and cut section of Verrucous carcinoma

**Fig. 2:**
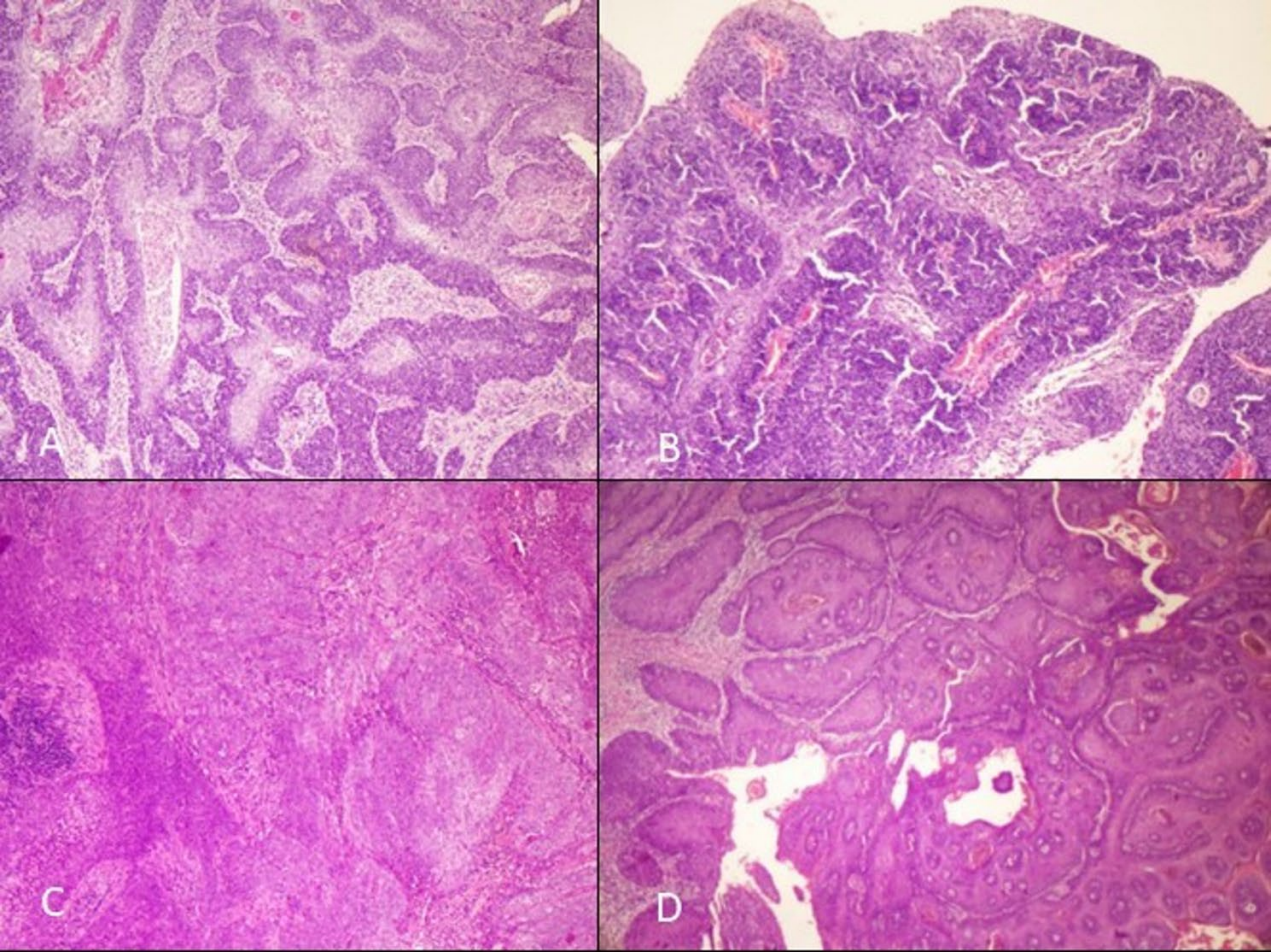
Microscopic images of HPV associated squamous cell carcinoma (H&E, 200x)- **A**- Basaloid **B**-Warty basaloid **C**- Lymphoepithelioma-like **D**- warty type

**Fig. 3:**
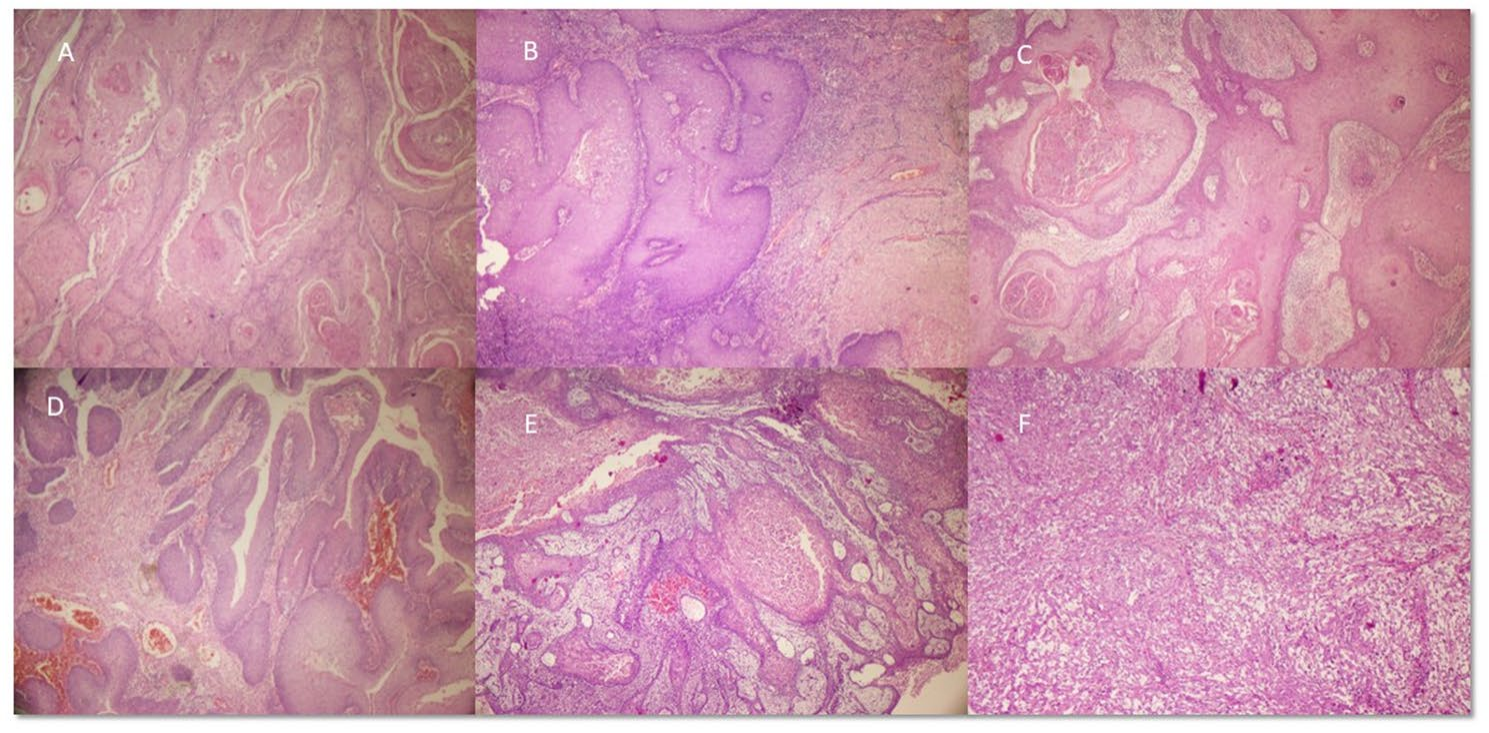
Microscopic images of HPV independent associated squamous cell carcinoma (H&E, 200x)- **A**-Usual, **B**- Verrucous, **C**- Usual (Carcinoma cuniculatum subtype) **D**- Papillary, **E**-Verrucous (Pseudoglandular subtype) **F**- Sarcomatoid type

Tumor differentiation showed that 50% of cases were well-differentiated (Grade 1), 41.7% were moderately differentiated (Grade 2), and 8.3% were poorly differentiated (Grade 3). The gross and microscopic images of penile SCC, HPV independent and associated depicted in Figs. 1, 2, and 3

Among the 59 cases with available pathological staging, pT1a was the most common stage (49.2%), followed by pT3 (20.3%), pT2 (13.6%), pTa (10.2%), pT1b (5.1%), and pTis (1.7%).

Lympho-vascular invasion was present in 10.2% of cases, while perineural invasion was seen in 15.6%. Immunohistochemical analysis for p16 expression, a surrogate marker of HPV association, was positive in 20.9% of tumors, while the majority (79.1%) were negative.The pathological characteristics are summarized in Table 2.

**Table 2:** Shows the pathological features of penile SCC

	Number	Percentage
Type of Specimen Small biopsy Wide local excision Partial penectomy Total penectomy	13 04 37 18	18% 5.5% 51.4% 25%
Histological subtype **A)HPV independent type**: Usual (including pseudoglandular subtype) Verrucous (including carcinoma cuniculatum) Papillary Sarcomatoid type **B)HPV-associated type**: Basaloid Warty Mixed (Warty Basaloid) Lymphoepithelioma like	55 8 2 1 2 2 1 1	76.4% 11.1% 2.8% 1.4% 2.8% 2.8% 1.4% 1.4%
Differentiation Grade 1 Grade 2 Grade 3	36 30 6	50.0% 41.7% 8.3%
Pathological stage (*n*> = 59) pTis pTa pT1a pT1b pT2 pT3	1 6 29 3 8 12	1.7% 10.2% 49.2% 5.1% 13.6% 20.3%
**Lymphovascular invasion**	6	10.2%
**Perineural Invasion**	9	15.6%
p16 IHC expression Negative Positive	57 15	79.1% 20.9%

**Fig. 4:**
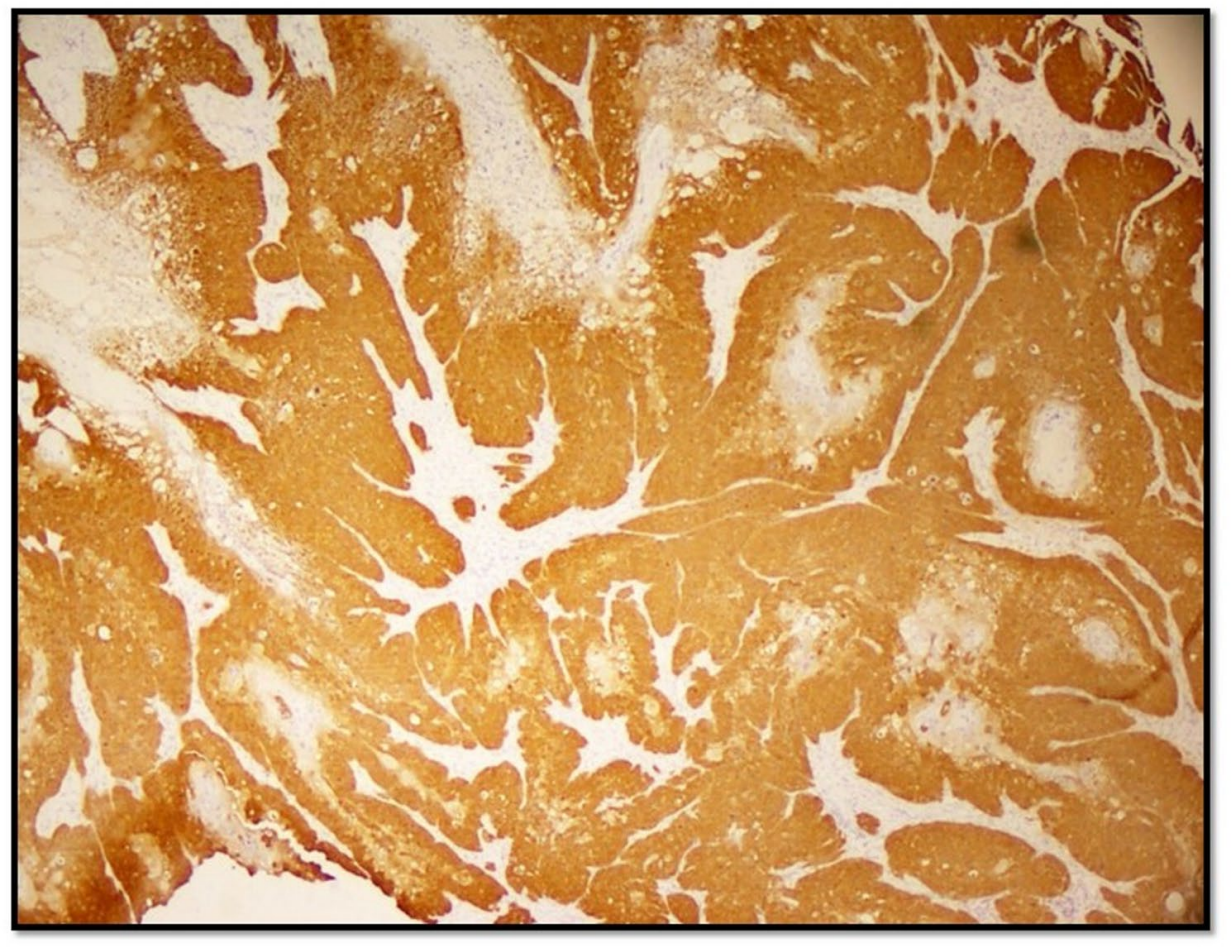
Shows p16 pattern Block positivity in Basaloid SCC on immunohistochemical staining (200x)

There was an agreement of 81.9% between HPV histological type of Penile SCC and p16 staining by immunohistochemistry. Block positivity for p16 immunohistochemistry shown in Fig 4.

Among the HPV-independent tumors (*n* = 66), the most common subtype was the usual type, comprising 83.6% of cases, of which 16.3% showed p16 positivity. Overall, 83.3% of HPV-independent tumors were p16-negative, while 16.7% were p16-positive. The correlation between usual-type histology and p16 negativity in this group was statistically significant (*p* < 0.05).

In contrast, among the HPV-associated tumors (*n* = 6), 66.7% were p16-positive. Basaloid and lymphoepithelioma-like carcinomas were consistently p16-positive (100%), while warty and papillary subtypes showed variable p16 expression (50% each). Only 33.3% of HPV-associated tumors were p16-negative. The association between basaloid histology and p16 positivity was also statistically significant (*p* < 0.05). The association of different histological types of HPV independent and associated SCC and p16 status by immunohistochemistry is shown in Table 3.

**Table 3:** Shows comparison of HPV associated and independent type of penile SCC on morphology with p16 immunostaining

HPV- Independent type On histology	P16 negative	p16 positive	*P* value	HPV- associated type On histology	p16 negative	p16 positive	*P* value
Usual type	46 (83.6%)	9 (16.3%)	< 0.05	Basaloid	0	2 (100%)	< 0.05
Verrucous	7 (87.5%)	1 (12.5%)		Warty	1 (50%)	1 (50%)	
Papillary	1 (50%)	1 (50%)		Lymphoepithelioma -like	0	1 (100%)	
Sarcomatoid	1 (100%)	0 (0.0%)		Warty- Basaloid	1(100%)	0 (0.0%)	
Total (66)	55 (83.3%)	11 (16.7%)		Total (6)	2 (33.3%)	4 (66.7%)	

The association of HPV-associated and HPV-independent squamous cell carcinoma (SCC) of the penis with pathological stage, inguinal lymph node involvement, and tumor grade, along with p16 expression status was studied ( Table 4).

There was no statistically significant difference in pathological staging between HPV-independent and HPV-associated SCC (*p* = 0.858), nor between p16-negative and p16-positive SCC (*p* = 0.349). In both groups, the majority of cases belonged to pT1a stage —47.3% in HPV-independent tumors and 75.0% in HPV-associated tumors. Similarly, 41.3% of p16-negative tumors and 76.9% of p16-positive tumors were staged as pT1a. Higher-stage tumors (pT2 and pT3) were observed slightly more in HPV-independent and p16-negative cases.

Inguinal lymph node metastasis was significantly more common in HPV-independent SCCs (*p* < 0.05) and in p16-negative tumors (*p* < 0.05). Among HPV-independent cases, lymph node involvement was present in 7 patients, compared to only 1 in the HPV-associated group. Similarly, lymph node metastasis was seen in 6 cases of p16-negative SCC and 2 cases of p16-SCC.

With respect to tumor differentiation, Grade 1 tumors (well-differentiated) were significantly more frequent in HPV-independent SCCs and p16-negative tumors (*p* < 0.05). All Grade 1 tumors were seen in the HPV-independent group and none in the HPV-associated group. Conversely, Grade 3 tumors (poorly differentiated) were more frequent in HPV-associated (5 cases) and p16-positive (5 cases) tumors, showing a statistically significant association (*p* < 0.05).

Follow up data was not available for majority of patients. 2 cases with distant metastasis and 2 cases of recurrence reported during follow-up occurring in usual type of SCC.

**Table 4:** Shows comparison between morphologic classification of penile carcinoma subtypes and p16 expression with pathological staging, lymph node status and histological grading

	HPV-independent SCC	HPV-associated SCC	*p* value	*P* 16-negative SCC	P16 positive SCC	*p* value
**Pathological staging (** ***n*** ** = 59)**						
pTis	1 (1.8%)	0 (0.0%)	0.858	1 (2.2%)	0 (0.0%)	0.349
pTa	6 (10.9%)	0 (0.0%)		5 (10.9%)	1 (7.7%)	
pT1a	26 (47.3%)	3 (75.0%)		19 (41.3%)	10 (76.9%)	
pT1b	3 (5.5%)	0 (0.0%)		3 (6.5%)	0 (0.0%)	
pT2	8 (14.5%)	0 (0.0%)		7 (15.2%)	1 (7.7%)	
pT3	11 (20.0%)	1 (25.0%)		11 (23.9%)	1 (7.7%)	
**Inguinal Lymph node status (** *n* ** = 59)**						
Present	7 (12.06%)	1 (25%)	< 0.05	6 (13.6%)	2 (13.3%)	< 0.05
Absent	5 (87.93%)	3 (75%)		38 (86.4%)	13 (86.7%)	
**Histological grading (** *n* ** = 72)**						
Grade 1	36 (54.5%)	0 (0%)	< 0.05	29 (48.3%)	6 (40%)	< 0.05
Grade 2	29 (43.9%)	1 (1.7%)		30 (50%)	4 (26.7%)	
Grade 3	1 (1.6%)	5 (83.3%)		1(1.7%)	5 (33.3%)	

## Discussion

Penile cancer is an uncommon malignancy representing less than 1% of all malignancies in the USA and Europe with higher incidence rates in developing countries like India [4]. It constituted 0.51% of all malignancies encountered during our 5-year study period.

Table 5 compares the clinicopathological features of Penile SCC in our study with other Indian and International studies [15–18].

**Table 5:** Comparison of clinicopathological features of penile SCC in our study and other Indian and international cohorts

	Our study	Moen et al.	Eich ML et al.	Júnior T et al.	Manipadam et al.
**Number of cases**	72	277	102	200	103
**Phimosis**	37.5%	-	-	70.5%	-
**Age**,** Mean** **Range**	58 years 31–89 years 58 years 31–89)	67 years	63 years	60s	26-81years
**Histological subtype**					
Usual	55 (76.4%)	165 (60%)	49 (48%)	79(35%)	78(75%)
Verrucous	8(11.1%)	20(3.6%)	1(1%)	-	4 (4%)
Papillary	2(2.8%)	6 (2.2%)	5(5%)	-	1 (1%)
Sarcomatoid	1(1.4%)	1(0.4%)	-	-	0%
Basaloid	2(2.8%)	31(11.2%)	17(17%)	8(4.0%)	1(1%)
Warty	2(2.8%)	22 (7.9%)	22(22%)	58(29.0%)	10(10%)
Warty basaloid	1(1.4%)	-	7(7%)	17(8.5%)	1(1%)
Lymphoepithelioma like	1(1.4%)	-	1(1%)	-	-
Mixed	-	41(15%)	-	32(16.0%)	8 (8%)
Others	-	-	-	6 (3.0%)	-
**HPV Histological type**					
HPV Independent type	91.7%	69.3%	53%	42%	80.5%
HPV Associated type	8.3%	30,7%	47%	58%	19.5%
**Tumor grade**					
Grade I	36 (50%)	-	18(18%)	25(12.5%)	-
Grade II	30 (41.7%)	-	57(56%)	77(38.5%)	-
Grade III	6 (8.3%)	-	27(26%)	98(49.0%)	-
**Tumor stage**					
T1	32 (61.5%)	137 (49%)	31 (30%)	45 (22.5%)	25 (24.3%)
T2	8 (15.4%)	97 (35%)	27 (27%)	53 (26.5%)	72(69.9%)
T3	12 (23.1%)	40 (14%)	41 (40%)	96 (48.0%)	6(5.8%)
T4	-	3 (3.1%)	3 (3%)	3.0%	-
Lymph node metastasis -Present	8 (13.6%)	94 (34%)	80(35%)	47(63.5%)	24 (23.3%)
LVI -Present	6 (10.2%)	94 (34%)	27 (26%)	71(35.5%)	-
**HPV DNA (PCR)**					
Positive	-	146 (53%)		91/113(80.5%)	-
Negative	-	131 (47%)	-	22/113(19.5%)	-
**p16 IHC IIHCimmunohistochemistry**					
Positive	15 (20.9%)	-	53 (52%)	128/173(74%)	-
Negative	57 (79.1%)	-	41 (40%)	45/173(26%)	-

The mean age at presentation in our study was 58 years, the youngest patient being 31 years old and the oldest patient was 87 years old. Glans penis was the most commonly affected site, typically presenting with an ulcero-proliferative lesion.

The percentage of HPV associated histological types were rare in our study accounting for (8.3%), while majority were HPV independent type (91.7%) Among the subtypes, Usual type of SCC constituted 76.3% followed by Verrucous carcinoma (11.1%), both of which are HPV-independent types. Among the HPV associated SCC, basaloid type was the most frequent. This correlated with a study by Manipadam et al. who observed 80.6% to be of HPV independent type [18]. In a recent study from India on 179 cases, 77% (137/179) were HPV-independent and HPV associated constituted 23% (42/179). The most common HPV-independent subtype was Usual SCC (72.6,whereas basaloid PSCC accounted for 50% of the HPV-associated cases in their study [19]. The usual type of SCC was most common subtype (48%) followed by Basaloid (20%) and Warty (14%) in a study by Cubilla et al. [13] This difference may be attributed to wide variation in infection with HPV and socioeconomic factors. PSCCs are known to occur through two distinct pathways of carcinogenesis, one related to human papillomavirus (HPV) infection and the other related to chronic inflammation [19]. The prevalence of phimosis in penile cancer reported ranged from 25–75% [20]. Phimosis was seen in 37.5% of our cases in our study. The low occurrence of HPV associated type of PSCC and frequent association of phimosis in our study probably suggests the second pathway of carcinogenesis related to chronic inflammation as the predominant factor in our study.

The proportion of penile cancer reported to be associated with high risk-HPV types ranges from 30 to 100%, depending on the population studied, based on the methods used for HPV detection and/or histological subtypes. The presence of HPV in tumour tissue can be detected using various methods, such as PCR amplification for the detection of HPV DNA. Based on the strong correlation between transcriptionally active HPV and p16INK4a overexpression in tumour cells, p16INK4a expression has been used as a surrogate marker of transforming HPV infections. Most penile cancers are SCC with frequent association with HPV infection, which can be diagnosed by tumor histology and confirmed by overexpression of p16 on IHC [14]. According to Cubilla et al., p16 overexpression is closely correlated with the presence of the HR-HPV genotype in penile cancer, as observed in cervical carcinoma and other HPV-related tumors [18]. 

Based on histology, 46% of tumors displayed an HPV-related subtype, and p16 IHC was positive in 52% of all cases with a *p* value of between histologic HPV associated subtype and p16 expression. p16 IHC also accurately predicted the presence of HPV in 25/26 (96%) of their cases [14]. This number is similar to a study by Olsen et al. who showed a pooled HPV DNA prevalence in 50.8% of penile cancers [7]. 

In our study, p16 immunostaining was negative in 79.2% (57/72) and positive in 20.8% (15/72). The concordance between histological subtype and p16 IHC was 81.9%. A higher concordance of p16 IHC with morphology was noted by Bilala et al. in their study [17]. 11 cases of the HPV independent type on histology (16.7%) showed p16 IHC positivity in our study. Olesen et al. noted HPV prevalence of 19.4% of histologically HPV-independent carcinomas similar to our study [7]. 

Our study not only proves positive staining of p16 in HPV- associated SCC, it also confirms the negative staining of p16 in HPV independent carcinomas.These findings are consistent with studies done by Cubilla et al. and Do et al. in penile carcinomas [18, 21]. p16 negativity does not necessarily indicate the absence of HPV infection. It may result from a true lack of HPV DNA, infection with low-risk HPV (LR-HPV), or other mechanisms such as p16 gene inactivation by promoter hypermethylation. p16 positivity is related to infection by only high risk-HPV DNA by molecular studies as shown in studies done by Cubilla et al. and Martins et al. [18, 23] However, it is also noted that some high-HPV associated lesions tend to be p16 negative [13]. This was interpreted in two ways: either due to presence of non-oncogenic, transient infection or inactivation of p16 gene by promoter hypermethylation. The presence of p16 overexpression was noted in 11 cases of histological independent type of SCC. Similar observation was noted by Cubilla et al. who suggested alternate pathways like pRB pathway which can result in p16 overexpression in the absence of HPV infection [18]. 

A significant challenge lies in the interpretation of p16 staining patterns, especially in determining the appropriate cut-off percentage of neoplastic cells to define positivity in penile carcinoma. Unlike cervical carcinoma, standardized criteria for p16 interpretation in penile SCC are lacking, leading to potential subjectivity and variability across studies [13]. Previous criteria for assessing p16 positivity have considered factors such as the staining location (nuclear and/or cytoplasmic), the percentage and distribution of positive cells, and the intensity of staining (weak, moderate, or strong). However, these criteria lack clear guidelines for setting cutoff values, leading to inherent subjectivity and potential disagreement in interpretation [13, 19]. In the current study, complete nuclear and cytoplasmic staining in all neoplastic cells was considered as p16 positive.

The majority of tumors in our study were Grade I and diagnosed at the T1 stage, with partial penectomy being the most frequently performed surgery. However, a recent study from India reported more advanced stage at presentation with pT2 (48%) or pT3 (30%) stages and nodal metastasis in 53.1%.

In our study, neither the histopathologic subtype (HPV-associated vs. HPV-independent) nor p16 expression showed a statistically significant association with pathological stage (*p* = 0.858 and 0.349, respectively). This finding is in concordance with studies by Bezerra et al., Martins et al. and Scheiner et al. [1, 22, 23] However, other studies have shown positive association between histopathologic subtypes and p16 overexpression with the pathological stage. Martins et al.., Djajadiningrat et al., Lont et al.. and Ferrandiz-Pulido et al. found presence of HPV DNA to have higher chance of survival than those without HPV DNA [22, 24–26]. In contrast to the studies mentioned above, some studies showed that high-risk HPV strains, particularly HPV16 and 18, were involved with aggressive subtypes, resulting in a worse survival rate than HPV independent penile cancer [23]. At present, there are no definitive prognostic or treatment differences between HPV-associated and HPV-independent penile tumors. However, recent evidence indicates that HPV-associated squamous cell carcinoma (SCC) may show a better response to radiation therapy, immunotherapy, or combined treatment approaches [2]. It has also been shown that determination of HPV status by PCR and p16 IHC do demonstrate benefits in survival in two recent meta analyses [27, 28]. 

Penile squamous cell carcinomas with TP53 mutations are found to be associated with a higher risk of lymph node metastases and poorer prognosis, and recent studies have proposed use of p53 immunohistochemistry as a reliable surrogate marker to mutational analysis in penile SCC that can help clinicians to better define risk groups and refine treatment strategies [29]. 

## Conclusion

In conclusion, our findings suggest that HPV-independent penile SCC, particularly the Usual subtype, is predominant in our cohort. The limited expression of p16 and frequent presence of phimosis support chronic inflammation as a major etiological pathway. While p16 remains a useful surrogate marker for HPV, variability in expression and lack of standardized interpretation criteria highlight the need for molecular confirmation in ambiguous cases. Our study is limited by its retrospective nature, small number of cases, lack of follow-up and use of p16 IHC which has inherent limitations with false negatives and positives when used alone to determine the HPV status.

Further multicenter studies are warranted to explore the prognostic significance of HPV status and its impact on treatment outcome in India. In view of discordance observed between histological subtype and the p16 expression, we recommend the molecular confirmation of HPV status by HPV DNA or RNA detection in future studies in India to validate p16 findings.

Efforts should concentrate on promoting male personal hygiene, educating about the risk factors for penile carcinoma, and emphasizing the importance of early detection and treatment.

## Data Availability

No datasets were generated or analysed during the current study.
